# Supplementary material for: Artificial Intelligence-Guided Analysis of WNT Pathway Alterations: Associations with Genomic Burden and Survival Across African American and Non-Hispanic White Populations, Age Groups, and FOLFOX Treatment in Colorectal Cancer
Source: Int J Mol Sci. 2026 Aug 8;27(16):7110. doi: 10.3390/ijms27167110 (PMC13513392; doi:10.3390/ijms27167110)
Supplement: Supplementary file 1 [file ijms-27-07110-s001.zip › ijms-4418519-supplementary.pdf]

Supplementary Information:

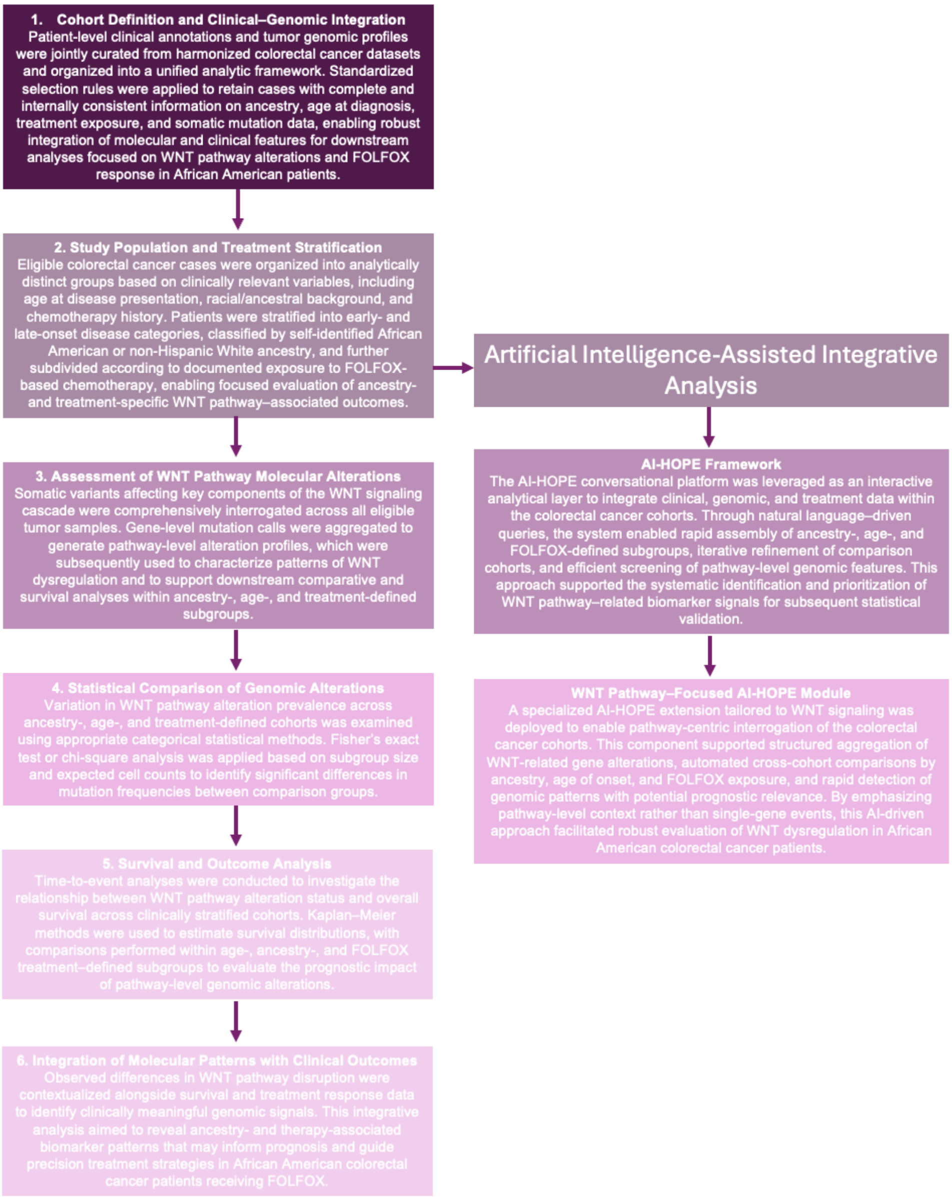

Figure S1. Overview of the AI-Driven Analytical Framework. This figure illustrates the integrated study pipeline used to investigate WNT pathway alterations in colorectal cancer. De-identified clinical, treatment, and genomic data were curated from large, publicly accessible CRC resources and harmonized into a unified analytic cohort. Patients were organized according to age at diagnosis (early- versus late-onset disease), self-reported ancestry (AA versus NHW), and exposure to FOLFOX chemotherapy. Pathway-level somatic alterations affecting WNT signaling were systematically quantified and compared across stratified groups using appropriate categorical statistical tests. Overall survival associations were subsequently examined using Kaplan-Meier methodology, enabling evaluation of ancestry- and treatment-specific prognostic effects of WNT pathway dysregulation within an artificial intelligence–assisted precision oncology framework.

Table S1 – Comparison of Early-Onset African American (AA) Patients Treated with FOLFOX versus Not Treated with FOLFOX

WNT Pathway			
Gene	Early-Onset AA<br>Treated with FOLFOX<br>n (%)	Early-Onset AA<br>Not Treated with FOLFOX<br>n (%)	p-value
**AMER1 Mutation**			
Present	1 (3.8%)	5 (6.7%)	1
Absent	25 (96.2%)	70 (93.3%)	
**APC Mutation**			
Present	20 (76.9%)	56 (74.7%)	1
Absent	6 (23.1%)	19 (25.3%)	
**AXIN1 Mutation**			
Present	1 (3.8%)	2 (2.7%)	1
Absent	25 (96.2%)	73 (97.3%)	
**AXIN2 Mutation**			
Present	0 (0.0%)	8 (10.7%)	0.1089
Absent	26 (100.0%)	67 (89.3%)	
**CTNNB1 Mutation**			
Present	0 (0.0%)	7 (9.3%)	0.1857
Absent	26 (100.0%)	68 (90.7%)	
**GSK3B Mutation**			
Present	0 (0.0%)	0 (0.0%)	1
Absent	26 (100.0%)	75 (100.0%)	
**RNF43 Mutation**			
Present	1 (3.8%)	5 (6.7%)	1
Absent	25 (96.2%)	70 (93.3%)	
**TCF7L2 Mutation**			
Present	5 (19.2%)	9 (12.0%)	0.5551
Absent	21 (80.8%)	66 (88.0%)	

Table S2 – Comparison of Late-Onset African American (AA) Patients Treated with FOLFOX versus Not Treated with FOLFOX

WNT Pathway			
Gene	Late-Onset AA<br>Treated with FOLFOX<br>n (%)	Late-Onset AA<br>Not Treated with FOLFOX<br>n (%)	p-value

**AMER1 Mutation**			
Present	5 (12.5%)	13 (7.6%)	0.487
Absent	35 (87.5%)	159 (92.4%)	
**APC Mutation**			
Present	27 (67.5%)	137 (79.7%)	0.1487
Absent	13 (32.5%)	35 (20.3%)	
**AXIN1 Mutation**			
Present	0 (0.0%)	5 (2.9%)	0.5862
Absent	40 (100.0%)	167 (97.1%)	
**AXIN2 Mutation**			
Present	1 (2.5%)	13 (7.6%)	0.477
Absent	39 (97.5%)	159 (92.4%)	
**CTNNB1 Mutation**			
Present	3 (7.5%)	13 (7.6%)	1
Absent	37 (92.5%)	159 (92.4%)	
**GSK3B Mutation**			
Present	0 (0.0%)	1 (0.6%)	1
Absent	40 (100.0%)	171 (99.4%)	
**RNF43 Mutation**			
Present	3 (7.5%)	19 (11.0%)	0.7733
Absent	37 (92.5%)	153 (89.0%)	
**TCF7L2 Mutation**			
Present	4 (10.0%)	15 (8.7%)	0.7625
Absent	36 (90.0%)	157 (91.3%)	

Table S3 – Comparison of Early-Onset Non-Hispanic White (NHW) Patients Treated with FOLFOX versus Not Treated with FOLFOX

**WNT Pathway**			
**Gene**	**Early-Onset NHW Treated with FOLFOX n (%)**	**Early-Onset NHW Not Treated with FOLFOX n (%)**	**p-value**
**AMER1 Mutation**			
Present	24 (6.4%)	22 (7.3%)	0.7633
Absent	351 (93.6%)	280 (92.7%)	
**APC Mutation**			
Present	291 (77.6%)	245 (81.1%)	0.304

Absent	84 (22.4%)	57 (18.9%)	
**AXIN1 Mutation**			
Present	6 (1.6%)	10 (3.3%)	0.2292
Absent	369 (98.4%)	292 (96.7%)	
**AXIN2 Mutation**			
Present	16 (4.3%)	16 (5.3%)	0.6553
Absent	359 (95.7%)	286 (94.7%)	
**CTNNB1 Mutation**			
Present	20 (5.3%)	23 (7.6%)	0.2928
Absent	355 (94.7%)	279 (92.4%)	
**GSK3B Mutation**			
Present	2 (0.5%)	5 (1.7%)	0.2516
Absent	373 (99.5%)	297 (98.3%)	
**RNF43 Mutation**			
Present	18 (4.8%)	20 (6.6%)	0.3919
Absent	357 (95.2%)	282 (93.4%)	
**TCF7L2 Mutation**			
Present	58 (15.5%)	60 (19.9%)	0.162
Absent	317 (84.5%)	242 (80.1%)	

Table S4 – Comparison of Late-Onset Non-Hispanic White (NHW) Patients Treated with FOLFOX versus Not Treated with FOLFOX

WNT Pathway			
**Gene**	**Late-Onset NHW Treated with FOLFOX n (%)**	**Late-Onset NHW Not Treated with FOLFOX n (%)**	**p-value**
**AMER1 Mutation**			
Present	71 (7.7%)	59 (9.0%)	0.4032
Absent	848 (92.3%)	594 (91.0%)	
**APC Mutation**			
Present	701 (76.3%)	478 (73.2%)	0.1836
Absent	218 (23.7%)	175 (26.8%)	
**AXIN1 Mutation**			
Present	18 (2.0%)	30 (4.6%)	**0.0045**
Absent	901 (98.0%)	623 (95.4%)	
**AXIN2 Mutation**			

Present	32 (3.5%)	64 (9.8%)	**4.44E-07**
Absent	887 (96.5%)	589 (90.2%)	
**CTNNB1 Mutation**			
Present	62 (6.7%)	38 (5.8%)	0.5239
Absent	857 (93.3%)	615 (94.2%)	
**GSK3B Mutation**			
Present	7 (0.8%)	7 (1.1%)	0.7092
Absent	912 (99.2%)	646 (98.9%)	
**RNF43 Mutation**			
Present	60 (6.5%)	96 (14.7%)	**1.48E-07**
Absent	859 (93.5%)	557 (85.3%)	
**TCF7L2 Mutation**			
Present	120 (13.1%)	118 (18.1%)	**0.0078**
Absent	799 (86.9%)	535 (81.9%)	

Table S5 – Comparison of Early-Onset versus Late-Onset African American (AA) Patients Treated with FOLFOX

WNT Pathway

Gene	Early-Onset AA Treated with FOLFOX n (%)	Early-Onset NHW Treated with FOLFOX n (%)	p-value
**AMER1 Mutation**			
Present	1 (3.8%)	24 (6.4%)	1
Absent	25 (96.2%)	351 (93.6%)	
**APC Mutation**			
Present	20 (76.9%)	291 (77.6%)	1
Absent	6 (23.1%)	84 (22.4%)	
**AXIN1 Mutation**			
Present	1 (3.8%)	6 (1.6%)	0.3768
Absent	25 (96.2%)	369 (98.4%)	
**AXIN2 Mutation**			
Present	0 (0.0%)	16 (4.3%)	0.613
Absent	26 (100.0%)	359 (95.7%)	
**CTNNB1 Mutation**			
Present	0 (0.0%)	20 (5.3%)	0.6306
Absent	26 (100.0%)	355 (94.7%)	
**GSK3B Mutation**			
Present	0 (0.0%)	2 (0.5%)	1
Absent	26 (100.0%)	373 (99.5%)	

**RNF43 Mutation**			
Present	1 (3.8%)	18 (4.8%)	1
Absent	25 (96.2%)	357 (95.2%)	
**TCF7L2 Mutation**			
Present	5 (19.2%)	58 (15.5%)	0.817
Absent	21 (80.8%)	317 (84.5%)	

Table S6 – Comparison of Early-Onset African American (AA) versus Early-Onset Non-Hispanic Whites (NHW) Patients Not Treated with FOLFOX

WNT Pathway			
**Gene**	**Early-Onset AA Not Treated with FOLFOX n (%)**	**Early-Onset NHW Not Treated with FOLFOX n (%)**	**p-value**
**AMER1 Mutation**			
Present	5 (6.7%)	22 (7.3%)	1
Absent	70 (93.3%)	280 (92.7%)	
**APC Mutation**			
Present	56 (74.7%)	245 (81.1%)	0.277
Absent	19 (25.3%)	57 (18.9%)	
**AXIN1 Mutation**			
Present	2 (2.7%)	10 (3.3%)	1
Absent	73 (97.3%)	292 (96.7%)	
**AXIN2 Mutation**			
Present	8 (10.7%)	16 (5.3%)	0.1498
Absent	67 (89.3%)	286 (94.7%)	
**CTNNB1 Mutation**			
Present	7 (9.3%)	23 (7.6%)	0.7999
Absent	68 (90.7%)	279 (92.4%)	
**GSK3B Mutation**			
Present	0 (0.0%)	5 (1.7%)	0.5877
Absent	75 (100.0%)	297 (98.3%)	
**RNF43 Mutation**			
Present	5 (6.7%)	20 (6.6%)	1
Absent	70 (93.3%)	282 (93.4%)	
**TCF7L2 Mutation**			
Present	9 (12.0%)	60 (19.9%)	0.1585
Absent	66 (88.0%)	242 (80.1%)	

Table S7 – Comparison of Late-Onset African American (AA) versus Late-Onset Non-Hispanic White (NHW) Patients Treated with FOLFOX

WNT Pathway			
Gene	Late-Onset AA Treated with FOLFOX n (%)	Late-Onset NHW Treated with FOLFOX n (%)	p-value
**AMER1 Mutation**			
Present	5 (12.5%)	71 (7.7%)	0.4265
Absent	35 (87.5%)	848 (92.3%)	
**APC Mutation**			
Present	27 (67.5%)	700 (76.2%)	0.287
Absent	13 (32.5%)	219 (23.8%)	
**AXIN1 Mutation**			
Present	0 (0.0%)	18 (2.0%)	1
Absent	40 (100.0%)	901 (98.0%)	
**AXIN2 Mutation**			
Present	1 (2.5%)	32 (3.5%)	1
Absent	39 (97.5%)	887 (96.5%)	
**CTNNB1 Mutation**			
Present	3 (7.5%)	62 (6.7%)	0.7482
Absent	37 (92.5%)	857 (93.3%)	
**GSK3B Mutation**			
Present	0 (0.0%)	7 (0.8%)	1
Absent	40 (100.0%)	912 (99.2%)	
**RNF43 Mutation**			
Present	3 (7.5%)	60 (6.5%)	0.7421
Absent	37 (92.5%)	859 (93.5%)	
**TCF7L2 Mutation**			
Present	4 (10.0%)	120 (13.1%)	0.8092
Absent	36 (90.0%)	799 (86.9%)	

Table S8 – Comparison of Late-Onset African American (AA) versus Late-Onset Non-Hispanic White (NHW) Patients Treated with FOLFOX

WNT Pathway			
Gene	Late-Onset AA Not Treated with FOLFOX n (%)	Late-Onset NHW Not Treated with FOLFOX n (%)	p-value
**AMER1 Mutation**			
Present	13 (7.6%)	59 (9.0%)	0.6464
Absent	159 (92.4%)	594 (91.0%)	
**APC Mutation**			
Present	137 (79.7%)	478 (73.2%)	0.1032

Absent	35 (20.3%)	175 (26.8%)	
**AXIN1 Mutation**			
Present	5 (2.9%)	30 (4.6%)	0.4448
Absent	167 (97.1%)	623 (95.4%)	
**AXIN2 Mutation**			
Present	13 (7.6%)	64 (9.8%)	0.4519
Absent	159 (92.4%)	589 (90.2%)	
**CTNNB1 Mutation**			
Present	13 (7.6%)	38 (5.8%)	0.5064
Absent	159 (92.4%)	615 (94.2%)	
**GSK3B Mutation**			
Present	1 (0.6%)	7 (1.1%)	1
Absent	171 (99.4%)	646 (98.9%)	
**RNF43 Mutation**			
Present	19 (11.0%)	96 (14.7%)	0.2681
Absent	153 (89.0%)	557 (85.3%)	
**TCF7L2 Mutation**			
Present	15 (8.7%)	118 (18.1%)	**0.0044**
Absent	157 (91.3%)	535 (81.9%)	

Table S9 – Comparison of Early-Onset African American (AA) versus Late-Onset AA (AA) Patients Treated with FOLFOX

**WNT Pathway**			
**Gene**	**Early-Onset AA Treated with FOLFOX n (%)**	**Late-Onset AA Treated with FOLFOX n (%)**	**p-value**
**AMER1 Mutation**			
Present	1 (3.8%)	5 (12.5%)	0.3904
Absent	25 (96.2%)	35 (87.5%)	
**APC Mutation**			
Present	20 (76.9%)	27 (67.5%)	0.5837
Absent	6 (23.1%)	13 (32.5%)	
**AXIN1 Mutation**			
Present	1 (3.8%)	0 (0.0%)	0.3939
Absent	25 (96.2%)	40 (100.0%)	
**AXIN2 Mutation**			
Present	0 (0.0%)	1 (2.5%)	1
Absent	26 (100.0%)	39 (97.5%)	
**CTNNB1 Mutation**			
Present	0 (0.0%)	3 (7.5%)	0.2727

Absent	26 (100.0%)	37 (92.5%)	
**GSK3B Mutation**			
Present	0 (0.0%)	0 (0.0%)	1
Absent	26 (100.0%)	40 (100.0%)	
**RNF43 Mutation**			
Present	1 (3.8%)	3 (7.5%)	1
Absent	25 (96.2%)	37 (92.5%)	
**TCF7L2 Mutation**			
Present	5 (19.2%)	4 (10.0%)	0.3009
Absent	21 (80.8%)	36 (90.0%)	

Table S10 – Comparison of Early-Onset African American (AA) versus Late-Onset AA (AA) Patients Not Treated with FOLFOX

WNT Pathway			
**Gene**	**Early-Onset AA Not Treated with FOLFOX n (%)**	**Late-Onset AA Not Treated with FOLFOX n (%)**	**p-value**
**AMER1 Mutation**			
Present	5 (6.7%)	13 (7.6%)	1
Absent	70 (93.3%)	159 (92.4%)	
**APC Mutation**			
Present	56 (74.7%)	137 (79.7%)	0.4813
Absent	19 (25.3%)	35 (20.3%)	
**AXIN1 Mutation**			
Present	2 (2.7%)	5 (2.9%)	1
Absent	73 (97.3%)	167 (97.1%)	
**AXIN2 Mutation**			
Present	8 (10.7%)	13 (7.6%)	0.5773
Absent	67 (89.3%)	159 (92.4%)	
**CTNNB1 Mutation**			
Present	7 (9.3%)	13 (7.6%)	0.8285
Absent	68 (90.7%)	159 (92.4%)	
**GSK3B Mutation**			
Present	0 (0.0%)	1 (0.6%)	1
Absent	75 (100.0%)	171 (99.4%)	
**RNF43 Mutation**			
Present	5 (6.7%)	19 (11.0%)	0.4037
Absent	70 (93.3%)	153 (89.0%)	
**TCF7L2 Mutation**			
Present	9 (12.0%)	15 (8.7%)	0.5711

Absent	66 (88.0%)	157 (91.3%)	

Table S11 – Comparison of Early-Onset Non-Hispanic White (NHW) versus Late-Onset Non-Hispanic White (NHW) Patients Treated with FOLFOX

WNT Pathway			
Gene	Early-Onset NHW Treated with FOLFOX n (%)	Late-Onset NHW Treated with FOLFOX n (%)	p-value
**AMER1 Mutation**			
Present	24 (6.4%)	71 (7.7%)	0.4764
Absent	351 (93.6%)	848 (92.3%)	
**APC Mutation**			
Present	291 (77.6%)	700 (76.2%)	0.6321
Absent	84 (22.4%)	219 (23.8%)	
**AXIN1 Mutation**			
Present	6 (1.6%)	18 (2.0%)	0.8362
Absent	369 (98.4%)	901 (98.0%)	
**AXIN2 Mutation**			
Present	16 (4.3%)	32 (3.5%)	0.6063
Absent	359 (95.7%)	887 (96.5%)	
**CTNNB1 Mutation**			
Present	20 (5.3%)	62 (6.7%)	0.4117
Absent	355 (94.7%)	857 (93.3%)	
**GSK3B Mutation**			
Present	2 (0.5%)	7 (0.8%)	1
Absent	373 (99.5%)	912 (99.2%)	
**RNF43 Mutation**			
Present	18 (4.8%)	60 (6.5%)	0.2906
Absent	357 (95.2%)	859 (93.5%)	
**TCF7L2 Mutation**			
Present	58 (15.5%)	120 (13.1%)	0.2926
Absent	317 (84.5%)	799 (86.9%)	

Table S12 – Comparison of Early-Onset Non-Hispanic White (NHW) versus Late-Onset Non-Hispanic White (NHW) Patients Not Treated with FOLFOX

WNT Pathway			
Gene	Early-Onset NHW Not Treated with FOLFOX n (%)	Late-Onset NHW Not Treated with FOLFOX n (%)	p-value
**AMER1 Mutation**			

Present	22 (7.3%)	59 (9.0%)	0.4366
Absent	280 (92.7%)	594 (91.0%)	
**APC Mutation**			
Present	245 (81.1%)	478 (73.2%)	**0.01**
Absent	57 (18.9%)	175 (26.8%)	
**AXIN1 Mutation**			
Present	10 (3.3%)	30 (4.6%)	0.4553
Absent	292 (96.7%)	623 (95.4%)	
**AXIN2 Mutation**			
Present	16 (5.3%)	64 (9.8%)	**0.0271**
Absent	286 (94.7%)	589 (90.2%)	
**CTNNB1 Mutation**			
Present	23 (7.6%)	38 (5.8%)	0.361
Absent	279 (92.4%)	615 (94.2%)	
**GSK3B Mutation**			
Present	5 (1.7%)	7 (1.1%)	0.6595
Absent	297 (98.3%)	646 (98.9%)	
**RNF43 Mutation**			
Present	20 (6.6%)	96 (14.7%)	**0.0006**
Absent	282 (93.4%)	557 (85.3%)	
**TCF7L2 Mutation**			
Present	60 (19.9%)	118 (18.1%)	0.5661
Absent	242 (80.1%)	535 (81.9%)	

Table S12. Spectrum of WNT Pathway Mutation Classes by Ancestry, Age at Diagnosis, and FOLFOX Exposure in Colorectal Cancer. This table details the relative distribution of somatic variant classes across core WNT pathway genes, stratified by ancestry (AA vs. non-Hispanic White), age of onset (early vs. late), and chemotherapy exposure (FOLFOX-treated vs. untreated). Reported categories include frameshift insertions and deletions, in-frame insertions and deletions, missense and nonsense variants, silent changes, splice site/region alterations, nonstop mutations, and translation start site variants. Percent values reflect the proportion of each mutation class within a given gene for the specified subgroup, enabling side-by-side comparison of mutational patterns across demographic and treatment-defined cohorts. Collectively, these data characterize how the composition of WNT pathway alterations varies by population context, disease timing, and therapy exposure.

	AA Samples				Non-Hispanic White Samples			
	Early-Onset		Late-Onset		Early-Onset		Late-Onset	
	Treated with FOLFOX	Not Treated with FOLFOX	Treated with FOLFOX	Not Treated with FOLFOX	Treated with FOLFOX	Not Treated with FOLFOX	Treated with FOLFOX	Not Treated with FOLFOX
**AMER1**								

Frame Shift Deletion	0.0%	0.0%	40.0%	14.3%	23.1%	6.3%	0.0%	14.1%
Frame Shift Insertion	0.0%	0.0%	0.0%	14.3%	7.7%	1.3%	0.0%	8.5%
Missense Mutation	0.0%	20.0%	0.0%	14.3%	19.2%	12.7%	0.0%	40.8%
Nonsense Mutation	100.0%	80.0%	60.0%	57.1%	50.0%	11.4%	0.0%	36.6%
**APC**								
Frame Shift Deletion	35.3%	33.0%	31.0%	27.4%	30.8%	28.0%	29.9%	26.8%
Frame Shift Insertion	11.8%	13.2%	16.7%	15.4%	9.4%	8.6%	10.4%	9.9%
In Frame Deletion	0.0%	0.0%	0.0%	0.0%	0.0%	0.0%	0.1%	0.1%
In Frame Insertion	0.0%	0.0%	0.0%	0.0%	0.0%	0.0%	0.0%	0.1%
Missense Mutation	5.9%	2.2%	0.0%	3.8%	6.7%	9.1%	2.7%	6.9%
Nonsense Mutation	44.1%	49.5%	52.4%	50.4%	51.4%	51.8%	54.8%	54.1%
Silent	0.0%	0.0%	0.0%	0.0%	0.0%	0.0%	0.1%	0.0%
Splice Region	0.0%	0.0%	0.0%	0.4%	0.0%	0.0%	0.1%	0.3%
Splice Site	2.9%	2.2%	0.0%	2.6%	1.9%	2.5%	1.9%	1.9%
**AXIN1**								
Frame Shift Deletion	0.0%	50.0%	0.0%	23.1%	0.0%	8.3%	22.7%	0.0%
Frame Shift Insertion	0.0%	0.0%	0.0%	0.0%	0.0%	0.0%	4.5%	0.0%
Missense Mutation	100.0%	50.0%	0.0%	53.8%	85.7%	83.3%	63.6%	0.0%
Nonsense Mutation	0.0%	0.0%	0.0%	15.4%	14.3%	8.3%	4.5%	0.0%
Splice Site	0.0%	0.0%	0.0%	7.7%	0.0%	0.0%	4.5%	0.0%
**AXIN2**								
Frame Shift Deletion	0.0%	50.0%	50.0%	50.0%	36.8%	33.3%	25.0%	22.5%
Frame Shift Insertion	0.0%	25.0%	50.0%	25.0%	10.5%	23.8%	20.0%	22.5%
In Frame Deletion	0.0%	0.0%	0.0%	0.0%	5.3%	0.0%	5.0%	1.4%
In Frame Insertion	0.0%	0.0%	0.0%	0.0%	0.0%	4.8%	0.0%	4.2%

Missense Mutation	0.0%	16.7%	0.0%	18.8%	31.6%	33.3%	42.5%	40.8%
Nonsense Mutation	0.0%	8.3%	0.0%	0.0%	15.8%	0.0%	2.5%	2.8%
Silent	0.0%	0.0%	0.0%	6.3%	0.0%	0.0%	0.0%	0.0%
Splice Site	0.0%	0.0%	0.0%	0.0%	0.0%	4.8%	5.0%	4.2%
Translation Start Site	0.0%	0.0%	0.0%	0.0%	0.0%	0.0%	0.0%	1.4%
**CTNNB1**								
Frame Shift Deletion	0.0%	0.0%	0.0%	0.0%	0.0%	0.0%	0.0%	2.4%
In Frame Deletion	0.0%	0.0%	0.0%	0.0%	0.0%	8.3%	10.1%	2.4%
Missense Mutation	0.0%	57.1%	66.7%	64.3%	52.4%	62.5%	62.3%	78.0%
Nonsense Mutation	0.0%	0.0%	0.0%	0.0%	4.8%	4.2%	1.4%	2.4%
Nonstop Mutation	0.0%	0.0%	0.0%	0.0%	0.0%	0.0%	1.4%	0.0%
Silent	0.0%	0.0%	0.0%	7.1%	0.0%	0.0%	0.0%	0.0%
Splice Site	0.0%	42.9%	33.3%	28.6%	42.9%	25.0%	24.6%	14.6%
**GSK3B**								
Frame Shift Insertion	0.0%	0.0%	0.0%	0.0%	0.0%	16.7%	0.0%	0.0%
Missense Mutation	0.0%	0.0%	0.0%	0.0%	100.0%	50.0%	57.1%	87.5%
Nonsense Mutation	0.0%	0.0%	0.0%	100.0%	0.0%	33.3%	28.6%	12.5%
Splice Site	0.0%	0.0%	0.0%	0.0%	0.0%	0.0%	14.3%	0.0%
**RNF43**								
Frame Shift Deletion	0.0%	70.0%	80.0%	57.7%	56.5%	56.0%	50.0%	61.3%
Frame Shift Insertion	0.0%	0.0%	20.0%	3.8%	13.0%	16.0%	15.8%	8.0%
Missense Mutation	100.0%	20.0%	0.0%	23.1%	13.0%	20.0%	15.8%	17.5%
Nonsense Mutation	0.0%	10.0%	0.0%	7.7%	13.0%	4.0%	15.8%	10.9%
Silent	0.0%	0.0%	0.0%	3.8%	0.0%	0.0%	0.0%	0.0%
Splice Site	0.0%	0.0%	0.0%	3.8%	4.3%	0.0%	1.3%	2.2%
Translation Start Site	0.0%	0.0%	0.0%	0.0%	0.0%	4.0%	1.3%	0.0%
**TCF7L2**								
Frame Shift Deletion	20.0%	44.4%	0.0%	35.3%	29.5%	28.4%	25.4%	38.8%

Frame Shift Insertion	0.0%	0.0%	0.0%	11.8%	18.0%	6.0%	7.7%	7.5%
In Frame Deletion	0.0%	0.0%	0.0%	0.0%	0.0%	1.5%	1.5%	2.2%
Missense Mutation	60.0%	33.3%	75.0%	17.6%	32.8%	38.8%	36.9%	36.6%
Nonsense Mutation	20.0%	11.1%	25.0%	29.4%	13.1%	13.4%	16.2%	9.7%
Splice Site	0.0%	11.1%	0.0%	5.9%	6.6%	10.4%	11.5%	4.5%
Translation Start Site	0.0%	0.0%	0.0%	0.0%	0.0%	1.5%	0.8%	0.7%

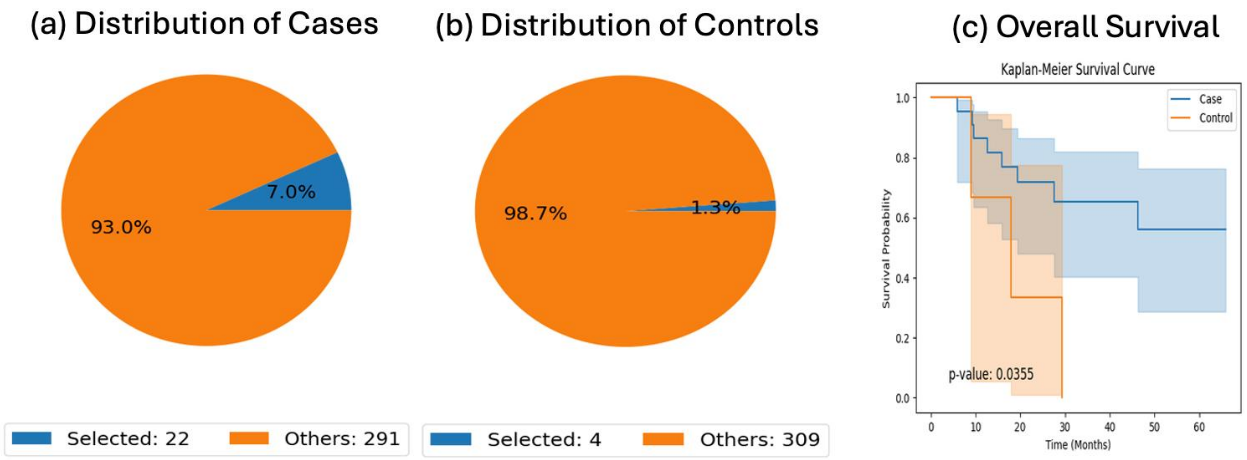

Figure S2. AI-enabled cohort definition and overall survival analysis of early-onset African American (AA) colorectal cancer patients treated with FOLFOX, stratified by WNT pathway alteration status. The AI-HOPE and AI-HOPE-WNT platforms were applied to construct molecularly and clinically defined case and control cohorts by integrating age at diagnosis, chemotherapy exposure, and WNT pathway alteration status. Panels (a) and (b) display pie charts summarizing the proportion of selected versus unselected samples within the case cohort, early-onset AA CRC patients treated with FOLFOX and harboring WNT pathway alterations (n = 22), and the control cohort, early-onset AA CRC patients treated with FOLFOX but lacking WNT pathway alterations (n = 4). Panel (c) presents a Kaplan–Meier analysis of overall survival, revealing a significant separation between groups, with WNT pathway–altered tumors exhibiting reduced survival compared with non-altered counterparts (log-rank p = 0.0355). Shaded regions denote 95% confidence intervals. Together, these results suggest that WNT pathway dysregulation may be associated with adverse

prognosis in early-onset AA CRC patients receiving FOLFOX and highlight the utility of AI-guided cohort stratification for identifying high-risk molecular subgroups.

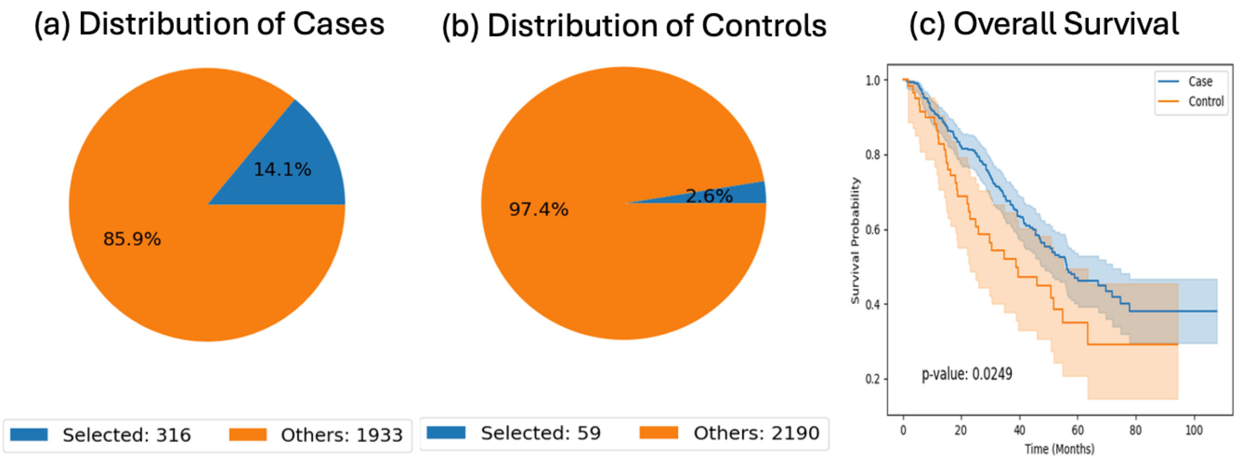

Figure S3. AI-driven cohort construction and overall survival analysis of early-onset Non-Hispanic White (NHW) colorectal cancer patients treated with FOLFOX, stratified by WNT pathway alteration status. Using the AI-HOPE and AI-HOPE-WNT analytical frameworks, patient cohorts were algorithmically defined by integrating age at diagnosis, chemotherapy exposure, and WNT pathway mutation status. Left panels display pie charts summarizing the proportion of selected versus unselected samples within the case cohort, early-onset NHW CRC patients treated with FOLFOX harboring WNT pathway alterations (n = 316), and the control cohort, early-onset NHW CRC patients treated with FOLFOX without WNT pathway alterations (n = 59). Right panel shows Kaplan–Meier estimates of overall survival, demonstrating a significant difference between groups, with WNT pathway–altered tumors associated with better survival outcomes compared with non-altered tumors (log-rank p = 0.0249). Shaded bands indicate 95% confidence intervals. These findings support a prognostic role for WNT pathway alterations in early-onset NHW CRC patients receiving FOLFOX and further illustrate the value of AI-assisted stratification for uncovering clinically relevant molecular risk groups.

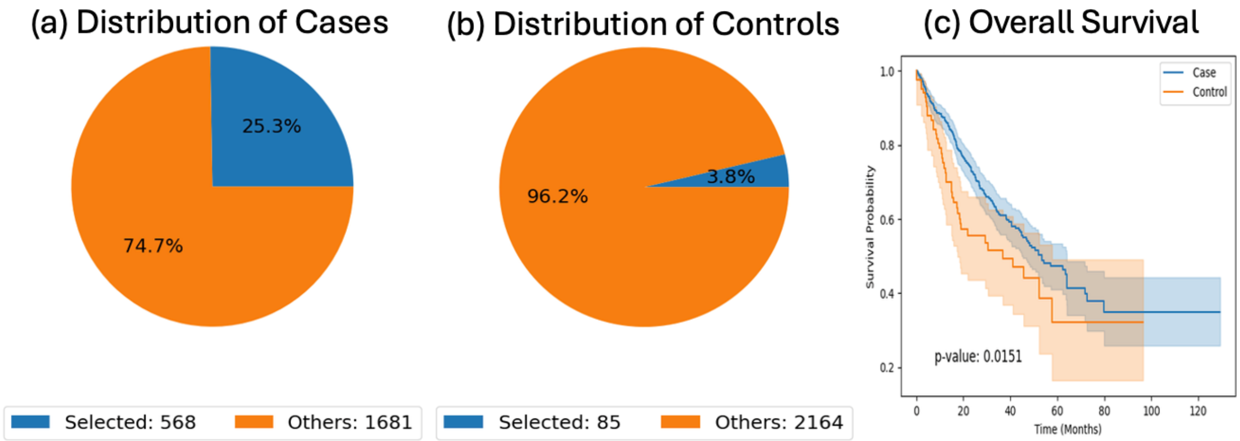

Figure S4. AI-assisted cohort stratification and overall survival analysis of late-onset Non-Hispanic White (NHW) colorectal cancer patients not treated with FOLFOX, according to WNT pathway alteration status. The AI-HOPE and AI-HOPE-WNT frameworks were employed to algorithmically identify case and control groups by integrating age at diagnosis, treatment exposure, and WNT pathway mutation status. Left panels depict pie charts summarizing the proportion of selected versus unselected samples within the case cohort, late-onset NHW CRC patients without FOLFOX treatment harboring WNT pathway alterations (n = 568), and the control cohort, late-onset NHW CRC patients without FOLFOX treatment lacking WNT pathway alterations (n = 85). Right panel shows Kaplan–Meier estimates of overall survival, demonstrating a statistically significant difference between groups, with WNT pathway–altered tumors associated with inferior survival outcomes compared with non-altered tumors (log-rank p = 0.0151). Shaded areas represent 95% confidence intervals. These results suggest that WNT pathway alterations may confer adverse prognostic significance in untreated late-onset NHW CRC and further underscore the value of AI-driven cohort selection for uncovering clinically meaningful molecular risk patterns.

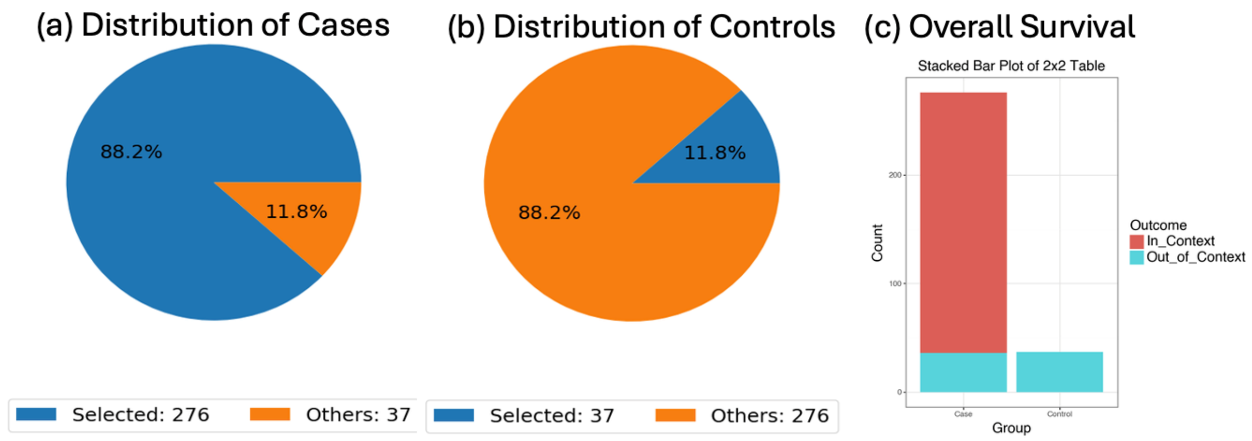

Figure S5. AI-assisted analysis of APC mutation enrichment in relation to WNT pathway alteration status across the African American (AA) colorectal cancer cohort. The AI-HOPE and AI-HOPE-WNT frameworks were applied to evaluate whether APC mutation frequency differs between AA CRC tumors with and without global WNT pathway alterations. Case and control cohorts were defined independent of treatment status, with the case group comprising tumors exhibiting WNT pathway alterations (n = 276) and the control group consisting of tumors without WNT pathway alterations (n = 37). Left panels show pie charts illustrating the proportion of selected (in-context) versus unselected samples within each cohort. Right panel presents a stacked bar plot summarizing APC mutation presence across groups, analyzed using a 2×2 contingency framework and Fisher's exact test. APC mutations were markedly enriched among WNT-altered tumors, with the odds ratio indicating a strong association between APC mutation status and WNT pathway dysregulation odds ratio (95% CI: 29.6–8213.7; $p < 0.001$). These findings reinforce APC as a core genomic driver underlying WNT pathway activation in AA CRC and demonstrate the utility of AI-guided comparative analytics for uncovering pathway-defining mutation relationships at the cohort level.
